# Anti-Inflammatory and Anti-Urolithiasis Effects of Polyphenolic Compounds from *Quercus gilva* Blume

**DOI:** 10.3390/molecules22071121

**Published:** 2017-07-05

**Authors:** Sung Hye Youn, Joo Hee Kwon, Jun Yin, Le Thi Tam, Hye Shin Ahn, Soon Chul Myung, Min Won Lee

**Affiliations:** 1 Laboratory of Pharmacognosy and Natural Product Derived Medicine, College of Pharmacy, Chung-Ang University, Seoul 156-756, Korea; lily6810@naver.com (S.H.Y.); yeye814@hanmail.net (J.H.K.); yinjun89@naver.com (J.Y.); letam18692@gmail.com (T.T.L.); hyaeshin@naver.com (H.S.A.); 2 Department of Urology, College of Medicine, Chung-Ang University, Seoul 156-756, Korea; uromyung@cau.ac.kr

**Keywords:** *Quercus gilva* Blume, anti-oxidation, anti-inflammation, polyphenol, anti-urolithiasis

## Abstract

*Quercus gilva* Bume (QGB, family Fagaceae) is a tall evergreen oak species tree that grows in warm temperate regions in Korea, Japan, China and Taiwan. *Quercus* plants have long been the basis of traditional medicines. Their clinical benefits according to traditional medicine include relief of urolithiasis, tremors and inflammation. In the present study, the anti-urolithiasis activity including anti-inflammatory and anti-oxidative activities, of some phenolic compounds isolated from QGB were described. Seven compounds were isolated and identified as picraquassioside D (**1**), quercussioside (**2**), (+)-lyoniresinol-9′α-*O*-β-d-xylopyranoside (**3**), (+)-catechin (**4**), (−)-epicatechin (**5**), procyanidin B-3 (**6**), and procyanidin B-4 (**7**). Compounds **5**–**7** showed potent anti-oxidative and anti-inflammatory activities. These compounds were further tested for their inhibition of the gene expression of the inflammatory cytokines. The three compounds **5**–**7** showed dose-dependent inhibitory activities on gene expression of COX-2 and IL-1β. In vivo, urolithiasis was induced more effectively in an animal model of acute urolithiasis by the administration of QGB extract. These results indicate the potential of compounds from QGB in the treatment of urolithiasis.

## 1. Introduction

Urolithiasis affects over 10~12% of the population in developed countries [1]. The disease has a detrimental effect on the healthcare system in part because of a recurrence rate of up to 50%. The recurrence of urinary stones together with the repetitive treatment cycles may eventually result in functional deterioration [2]. Recurrence increases the risk of subsequence relapse and the interval between relapses often become shorter [3,4]. Factors that contribute to relapse include young age of the first occurrence, family history and the presence of infection stones. 

The treatment of urinary stones varies depending on the stone size and location. Stones that are too large to pass through the urinary tract must be broken into smaller pieces so that they can pass through [4]. The procedures used to this end include extracorporeal shock wave lithotripsy (ESWL), uretroscopy (URS), and percutaneous nephrolithotomy (PNL) [5]. However, the removal of stones by these procedures does not reduce the risk of reoccurrence of urinary stones. Moreover, ESWL may cause serious side effects including renal damage, hypertension and renal function impairment [6].

While some medical treatments benefit the clearance of urinary stones, an ideal clinical drug treatment should prevent urinary stones from reoccurring [7]. No satisfactory drug is yet available.

With the aim of finding a truly beneficial drug for urolithiasis, the traditional use of plants to treat stone diseases may be instructive [8]. 

*Quercus* Genus can be classified to four categories, including *Fagaceae*, *Castanopis*, *Castanea* and *Quercus* species. Among the four categories, *Quercus* specie have the greatest number of plants (32 species). *Quercus* plants can also be divided to two types, which are deciduous and evergreen broad-leaved plants. The most common deciduous broad-leaved trees are *Quercus mongolica*, *Q. serrata*, *Q. dentata*, *Q. acutissima*, and *Q. variabilis*. The most common evergreen broad-leaved trees are *Q. gilva*, *Q. acuta*, *Q. glauca*, *Q. Ilex*, and *Q. salicina* [9,10]. *Quercus gilva* Bume (QCB) of the family Fagaceae is a tall evergreen tree distributed in the lowland mountain regions of Jeju Island in Korea. QGB is an oak species found in warm temperate regions in Japan, China and Taiwan [11,12].

As well as being the basis of a traditional medicine, clinical effects ascribed to QGB include the relief of urolithiasis and tremor, cessation of bleeding, local anesthesia, fever control, and inhibition of inflammation, oxidation, and infections caused by *Candida*, bacteria, and fungi [13,14,15,16]. 

Several phytochemical and biological studies revealing anti-oxidation and anti-diabetic activities of QGB have been performed, with triterpene and phenolic compounds reported from the flowers and bark, respectively [17,18]. However, anti-inflammatory activity, inhibition of cytokines and anti-urolithiasis activity of *Quercus gilva* (QG) have not yet been examined in detail. The present study assessed the potential benefit of QGB and phenolic compounds isolated from QGB against urolithiasis, including their anti-inflammatory and anti-oxidative activities. 

## 2. Results and Discussion

**Figure 1:**
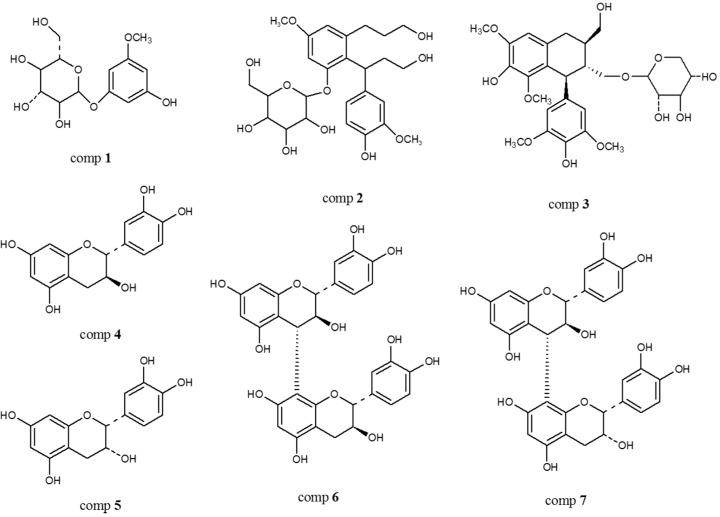
Structures of QGB compounds **1**–**7**.

Seven isolated compounds were identified as picraquassioside D (**1**, 8.0 mg) [18], quercussioside (**2**, 295.2 mg) [19], (+)-lyoniresinol 9′α-*O*-β-d-xylopyranoside (**3**, 746.2 mg) [20], (+)-catechin (**4**, 1.62 g) [21], (−)-epicatechin (**5**, 902.8 mg) [22], procyanidin B-3 (**6**, 186.3 mg) [23], and procyanidin B-4 (**7**, 1.04 g) [24] by comparing their spectral data with values reported in the literature (Figure 1).

Free radicals and other reactive oxygen species are generated by exogenous chemicals or endogenous metabolic processes in food systems or the human body. These radicals may cause oxidative damage resulting in cell death and tissue damage by oxidizing biomolecules [25]. Oxidative damage plays a significant pathological role in human disease. As examples, atherosclerosis, cancer, emphysema, cirrhosis, and arthritis have all been correlated with oxidative damage [25,26]. Therefore, ingestion of anti-oxidative supplements or foods containing anti-oxidants can be an important way to reduce oxidative damage [27]. Recently, natural anti-oxidants including vitamins C and E, sesamol, and carnosic acid have been introduced in foods in response to consumer demand. Presently, the anti-oxidative activity of compounds from QGB was explored by assessing their DPPH radical and NBT superoxide scavenging activities. DPPH easily accepts hydrogen because of the presence of the nitrogen of hydrazyl moiety. This allows anti-oxidative activity to be measured based on the loss of the violet color of DPPH following the incorporation of the hydrogen [28]. NBT is able to easily accept superoxide because of its unstable anionic nature. Similar to DPPH, the anti-oxidative activity of NBT is indicated by the loss of the yellow color [29].

DPPH radical scavenging activities were tested with the treatment of samples from various parts of QGB plants. Leaves (19.15 ± 0.22 μg/mL), branch (33.07 ± 0.34 μg/mL), bark (10.58 ± 0.85 μg/mL), and wood (16.14 ± 0.80 μg/mL) of 80% acetone QG, exhibited a similar degree of free radical scavenging activity compared with the positive control, l-ascorbic acid (6.04 ± 0.27 μg/mL), with the bark fraction displaying the most potent DPPH radical scavenging activity (Table 1). DPPH radical scavenging activities were also tested following treatment of each compound isolated from QGB. DPPH radical scavenging activity was evident for IC_50_ values of compounds **3** (74.23 ± 0.39 μM), **4** (43.37 ± 0.57 μM), **5** (28.80 ± 1.02 μM), **6** (15.10 ± 0.34 μM) and **7** (12.15 ± 0. 18 μM). **4**–**7** exhibited potent DPPH radical scavenging activities [**4** (43.37 ± 0.57 μM), **5** (28.80 ± 1.02 μM), **6** (15.10 ± 0.34 μM) and **7** (12.15 ± 0. 18 μM)]. In particular, **6** and **7** were more potent than the l-ascorbic acid positive control (IC_50_ = 27.41 ± 1.28 μM) (Table 1).

NBT/superoxide scavenging activities were tested with the material obtained from leaves, branch, bark, and wood. The activities of different parts of *Quercus gilva* were 7.50 ± 0.47, 20.07 ± 2.24, 4.43 ± 0.16, and 7.07 ± 0.98 μg/mL, respectively, compared to the activity of the allopurinol positive control (1.08 ± 0.22 μg/mL) (Table 2). Scavenging activities of the isolated compounds were tested. Compounds **1**–**3** did not exhibit activity. The activities of compounds **4**, **5**, **6,** and **7** were (15.44 ± 1.44, 11.01 ± 0.16, 7.21 ± 0.38 μM, and 7 8.67 ± 0.22 μM, respectively. Compounds **6** and **7** exhibited especially potent NBT/superoxide scavenging activities, with IC_50_ values of 7.21 ± 0.38 μM and 8.67 ± 0.22 μM, respectively, compared with the allopurinol control (IC_50_ = 5.43 ± 0.40 μM) (Table 2).

NO is generated in different cell types by at least three isoforms of NOS. Neuronal NOS (nNOS) and endothelial NOS (eNOS) are constitutively expressed and their enzymatic activity is Ca^2+^/calmodulin-dependent. The third enzyme is an inducible and Ca^2+^-independent isoform of NOS (iNOS) that is expressed in virtually all cell types after stimulation with LPS and/or with different cytokines, such as interferon-gamma (IFN-γ), interleukin-1-beta (IL-1β), or tumor necrosis factor-alpha (TNF-α). Massive amounts of NO produced by iNOS under pathological conditions (e.g., acute and chronic inflammation) are potentially harmful, especially when time-spatial regulation of iNOS expression becomes compromised. During inflammation associated with different pathogens, NO production increases significantly and may become cytotoxic [30]. In addition, the over-production of NO has also been also implicated in inflammatory skin disease [31,32]. Moreover, the free radical nature of NO and its high reactivity with oxygen to produce peroxynitrite (ONOO-) makes NO a potent pro-oxidant molecule capable of inducing oxidative damage and potential harm towards cellular targets [33]. Thus, inhibition of NO production in response to inflammatory stimuli might be a useful therapeutic strategy in inflammatory diseases [34,35]. To examine the effect of QGB on LPS-induced NO production in RAW 264.7 cells, cells were treated fractions from QGB and then untreated or exposed to LPS (1 μg/mL) for 20 h. Compounds **1**, **2**, and **4** did not inhibit NO production. However, potent inhibition of NO production was produced by compounds **3** (9.14 ± 0.45 μM), **5** (14.47 ± 5.29 μM), **6** (7.07 ± 0.40 μM), and **7** (1.44 ± 1.03 μM) compared with l-NMMA (IC_50_ = 2.72 ± 0.80 μM). (Table 3).

**Figure 2:**
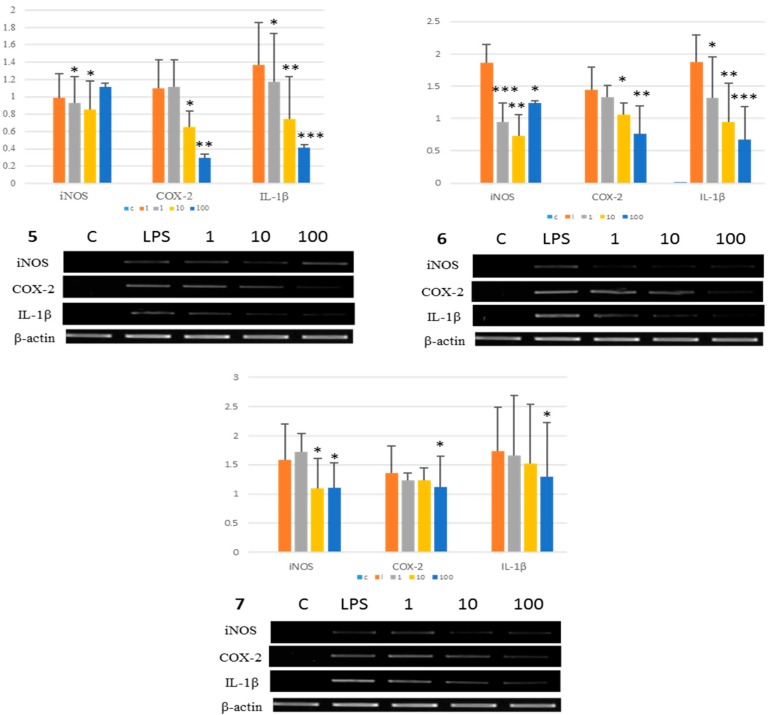
Inhibition of mRNA expression of iNOS, COX-2 and IL-1β by phenolic compounds **5**–**7** from QGB. Activities were determined by RT-PCR at least three times. Concentration of LPS was 1 μM. Values bearing different superscripts in same columns are significantly different (*: *p* < 0.05, **: *p* < 0.01, ***: *p* < 0.001) (*n* = 3).

Urolithiasis has been linked to many functional genes, including urokinase, vitamin D receptor gene, and especially calcium-sensing receptor gene (CaSR) [36,37,38]. Proinflammatory cytokines, such as IL-1β, markedly increase CaSR mRNA levels through a response mediated by nuclear factor-kappa B (NF-κB) [39]. Moreover, iNOS and COX-2 are induced by IL-1β. Thus, inhibition of iNOS, COX-2, and IL-1β expression may be therapeutic for urolithiasis [40,41,42]. Compounds **5**–**7**, which displayed potent anti-oxidative and anti-inflammatory activities, were selected to measure their inhibitory activities on gene expression of the related inflammatory cytokines iNOS, COX-2, and IL-1β. All three compound **5**–**7** showed dose-dependent inhibitory activities on gene expression of COX-2 and IL-1β. Compounds **6** and **7** inhibited gene expression of iNOS (Figure 2).

**Figure 3:**
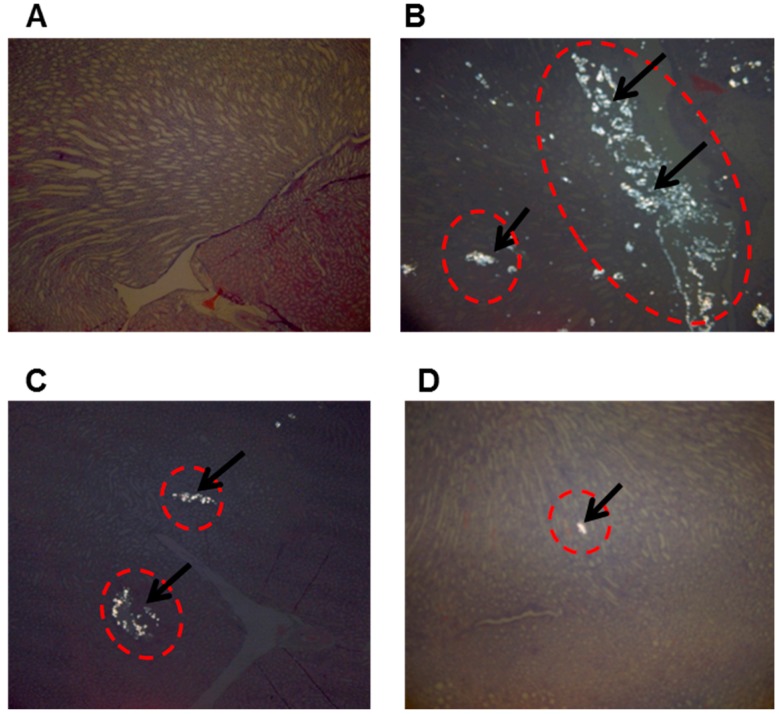
Representative microscopic images of stained of kidney sections from X-rats. The panels are as follows: blank (normal) (**A**); control (0.4% of ethylene glycol) (**B**); EG + low dose of drug (**C**); EG + high dose of drug (**D**). Arrows mean stones of kidney from X-rat. All panels are sections viewed by polarized light microscope (100×) after Hematoxylin and Eosin staining and under light microscope after Pizzolato’s staining (40×), respectively.

In our in vivo study results, urolithiasis was induced more effectively in 0.4% EG of the acute model than 0.2% EG in the acute and chronic models (Table 4). The present study provides evidence that IP of EG + low drug (50 mg/kg) and EG + high drug (100 mg/kg) of the QGB extract containing a rich polyphenol content inhibits the development of urolithiasis in the animal. In addition, the stone values for upper pole, mid pole and lower pole of the kidney on each section are shown in Table 5 and Figure 3. The results showed that QGB possessed a potent inhibitory activity in both the low drug group (2.33 mg) and high drug group (12.17 mg), compared with the negative control group (119.33 mg) (Table 5). The representative stained microscopic images of kidney tissue (Figure 3) showed similar results to Table 5. (+)-Catechin and (−)-epicatechin are well known potent anti-oxidation and anti-inflammation agents, and it was reported that they decreased calcium concentration significantly [43]. Moreover, red and white grape seed extract, where catechin, epicatechin and procyanidin dimers B1 to B4 are major phenolic components in the extract, were reported to show potent prevention of stone formation [44]. Therefore QGB, which contains abundant catechin and procyanidins may be effective to treat urolithiasis.

## 3. Materials and Methods

### 3.1. Plant Material

QGB (7.0 kg) was collected from the Halla Arboretum, Jeju, Republic of South Korea, April 2009, and was certificated by arboretum chief Dr. Kim Chul Soo. It was labeled “QG2009-04” and preserved as a voucher specimen.

### 3.2. Instruments

The stationary phases for the column chromatographic isolation were Sephadex LH-20 (10–25 μm; GE Healthcare Bio-Science AB, Uppsala, Sweden), MCI-gel CHP 20P (75–150 m; Mitsubishi Chemical, Tokyo, Japan) and ODS-B gel (40–60 m; Daiso, Osaka, Japan). The ODS-B gel was used as the stationary phase for medium-pressure liquid chromatography (MPLC). A Waters 650E sample detector (Waters, Seoul, Korea), 110UV/VIS detector (Gilson, Middleton, WI, USA) and model TBP5002 pump (Tauto Biotech, Sanghai, China) were used. Thin layer chromatography (TLC) was carried out using a pre-coated silica gel 60 F254 plate (Merck, Darmstadt, Germany) with chloroform, methanol and water (70:30:4 by volume). Spots were detected under ultraviolet (UV) radiation (254 nm) and by spraying with FeCl_3_ and 10% H_2_SO_4_ or anisaldehyde-H_2_SO_4_ followed by heating. 

One-dimensional ^1^H- (300 or 600 MHz) and ^13^C- (75 or 150 MHz) nuclear magnetic resonance (NMR) experiments were recorded with Gemini 2000 and VNS devices (Varian, Palo Alto, CA, USA) at the Center for Research Facilities, Chung-Ang University. Low-resolution fast atom bombardment mass spectroscopy (LRFAB-MS) was done using a JMSAX505WA apparatus (JEOL, Tokyo, Japan) at the National Center for Inter-University Research Facilities, Seoul National University. 

### 3.3. Extraction and Isolation of QGB Compounds

**Figure 4:**
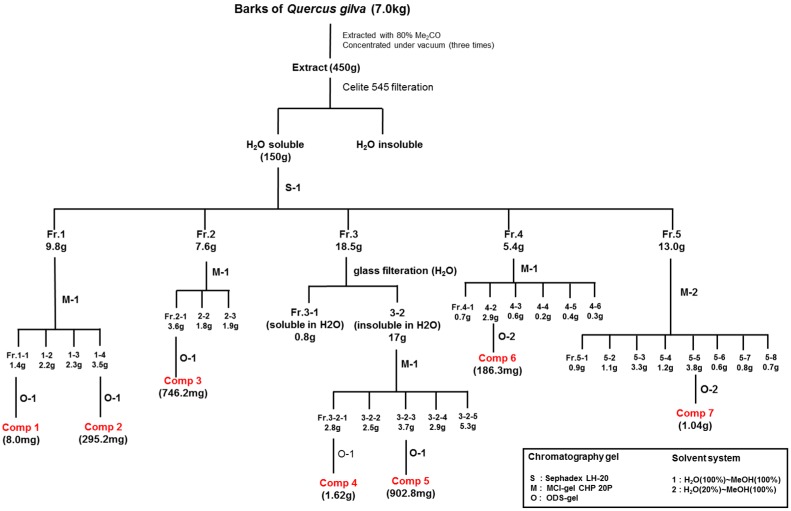
Extraction and separation processes of active compounds from bark of *Quercus gilva* Blume.

The QGB material (7.0 kg) was extracted for 72 h at room temperature with 80% aqueous acetone. After removing the acetone under vacuum, the aqueous solution was filtered through filter paper (Tokyo Roshi Kaisha Ltd., Tokyo, Japan), and re-filtered using the Celite 545. The filtrate was concentrated and applied to a Sephadex LH-20 column (10–25 μm), and eluted with water containing increasing proportions of methanol to afford five fractions (Figure 4): fraction 1 (9.8 g), fraction 2 (7.6 g), fraction 3 (18.5 g), fraction 4 (5.4 g), and fraction 5 (13.0 g). Repeated column chromatography of fraction 1 (9.8 g) on MCl-Gel CHP 20P (75–150 μm, 5 cm × 80 cm) using a water − methanol gradient yielded another four semi-fractions (fraction 1-1~1-4) and then fraction 1-1 (1.4 g) on ODS-B gel chromatography (40–60 cm) using a water − methanol gradient yielded [1-β-d-gluco-pyranosyloxy-3-methoxy-5-hydroxybenzene (picraquassioside D)] (**1**, 8.0 mg, Figure 4). Repeated column chromatography of fraction 1-4 (3.5 g) on an ODS-B gel (40–60 m; Daiso) using a water − methanol gradient yielded quercussioside (**2**, 295.2 mg, Figure 4).

Repeated column chromatography of fraction 5 (13.0 g) on MCl-Gel CHP 20P (75–150 μm, 5 cm × 80 cm) using a water (20%) − methanol (100%) gradient yielded eight semi-fractions (5-1~5-8) and repeated column chromatography fraction 5-5 (3.8 g) on ODS-B gel (40–60 m) using a water (20%) − methanol (100%) gradient yielded Procyanidin B-4 (**7**, 1.04 g, Figure 4). 

Repeated column chromatography of fraction 2 (7.6 g) on MCl-Gel CHP 20P (75–150 μm, 5 cm × 80 cm) and ODS-B gel (40–60 m) using a water − methanol gradient yielded [(+)-lyoniresinol 9′α-*O*-β-d-xylopyranoside] (**3**, 746.2 mg, Figure 4). 

Column chromatography of fraction 3 (18.5 g) on MCl-Gel CHP 20P (75–150 μm, 5 cm × 80 cm) using a water − methanol gradient yielded five semi-fractions (3-1~3-5). Repeated column chromatography of fraction 3-1 (2.8 g) and 3-3 (3.7 g) and ODS-B gel (40–60 m) using a water − methanol gradient yielded [(+)-catechin] (**4**, 1.62 g) and [(−)-epicatechin] (**5**, 902.8 mg, Figure 4).

Repeated column chromatography of fraction 4 (5.4 g) on MCl-Gel CHP 20P (75–150 μm, 5 cm × 80 cm) using a water − methanol gradient yielded six semi-fractions (4-1~4-6) and repeated column chromatography fraction 4-2 (2.9 g) on ODS-B gel (40–60 m) using a water (20%) − methanol (100%) gradient yielded procyanidin B-3 (**6**, 186.3 mg, Figure 4). 

### 3.4. Measurement of 2,2-Diphenyl-1-picrylhydrazyl (DPPH) Radical Scavenging Activity

Anti-oxidant activity was determined on the basis of the scavenging activity of the stable DPPH free radical (Sigma-Aldrich, St. Louis, MO, USA) [29]. Twenty microliter aliquots in absolute ethanol were added to 180 μL of 0.1 mM DPPH in absolute ethanol. After mixing gently and standing for 30 min, the optical density was measured at 518 nm using an ELISA reader (TECAN, Salzburg, Austria). The free radical scavenging activity was calculated as inhibition rate (%) = [1 − (sample optical density [OD]/control OD)] × 100 and IC_50_ values, defined as the concentration that could scavenge 50% DPPH free radical. l-ascorbic acid was used as positive control.

### 3.5. Measurement of Nitroblue Tetrazolium (NBT)/Superoxide Scavenging Activity

The NBT method was applied using a proper modification of a previously published method [29]. A reaction mixture with a final volume of 632 μL in an Eppendorf tube was prepared with 50 mM phosphate buffer (pH 7.5) containing EDTA (0.05 mM), hypoxanthine (0.2 mM), 63 μL NBT (1 mM; Sigma-Aldrich), 63 μL of aqueous or ethanolic extract (distilled water for the control), and 63 μL of xanthine oxidase (1.2 U/μL, Sigma-Aldrich). The xanthine oxidase was added last. For each sample, a blank was included. The subsequent rate of NBT reduction was determined on the basis of sequential spectrophotometric determinations of absorbance at 612 nm. The solutions were prepared daily and kept from light. The results are expressed as the percentage inhibition of the NBT reduction with respect to the reaction mixture without sample (buffer only). Superoxide anion scavenging activities were calculated as [1 − (sample OD − blank OD)/(control OD − blank OD)] × 100 and are expressed IC_50_ values, which were defined as the concentrations at which 50% of NBT/superoxide anion were scavenged. Allopurinol (Sigma-Aldrich) was used as positive control.

### 3.6. Cell Culture

RAW 264.7 murine macrophage cell line was purchased from the Korean Cell Line Bank (Seoul, South Korea). The cells were grown in culture flasks containing Dulbecco’s Modified Eagle’s Medium (DMEM; Sigma-Aldrich) supplemented with 10% fetal bovine serum (FBS) and antibiotics (100 IU/mL penicillin G and 100 mg/mL streptomycin; Gibco BRL, Grand Island, NY, USA). Cell cultures were cultured at 37 °C in a humidified atmosphere of 5% CO_2_.

### 3.7. Nitric Oxide (NO) Production and NO Inhibitory Activity

Experimental NO method was done using a appropriate modification of published method [45]. NO is associated with many conditions, including inflammation. The production of NO was assayed by measuring nitrite in the RAW 264.7 macrophage culture supernatant. Cells were seeded at a density 3 × 10^6^ cells/mL in wells of 96-well culture plates. After pre-incubation for 2~3 h, the cells were treated with compounds (1, 10, 50 and 100 μM) and then stimulated with lipopolysaccharide (LPS, 1 μg/mL) for 20 h. The LPS treated cells produced approximately 2.5-fold more NO than the control cells. The cell culture medium was harvested and used to determine NO level using the Griess reaction. For this, the supernatant was mixed with an equal volume of Griess reagent (1% sulfanilamide, 0.1% naphthylethylenediamine dihydrochloride, and 2.5% phosphoric acid) and incubated at room temperature for 10 min. The concentration of nitrite was determined by measuring the absorbance at 540 nm using an ELISA reader (TECAN, Salzburg, Austria) and comparing the values to a standard curve generated using sodium nitrite (NaNO_2_). NO production inhibitory activity was calculated as inhibition rate (%) = [1 − (sample OD − blank OD)/(control OD − blank OD)] × 100. The IC_50_ value (concentration that inhibited 50% of NO production) was determined.

### 3.8. Reverse Transcription-Polymerase Chain Reaction (RT-PCR)

Total RNA was isolated from tissue using 1 mL of TRIzol reagent (Invitrogen, Carlsbad, CA, USA) that was added to cultured plate. Cells were homogenized and transferred to a centrifuge tube. After fter 5 min at room temperature, chloroform was added (0.2 mL/mL TRIzol reagent), the tube was shaken vigorously by hand for 15 s, and then incubated at 15 °C to 30 °C for 3 min. The homogenate was centrifuged at 12,000 rpm at 4 °C for 15 min. The upper aqueous phase was transferred to a fresh tube and an equal volume of 2-propanoCl was added. After incubation at 4 °C for 15 min, the sample was centrifuged as before. The supernatant was removed and the pellet was washed using 500 μL of 70% ethanol followed by centrifugation as before. The pellet of purified RNA was briefly dried then dissolved in diethyl pyrocarbonate-distilled water (DEPC-DW). Total cellular RNA was reverse transcribed at 42 °C for 30 min in a containing reverse transcriptase (TaKaRa, Shiga, Japan), 10× buffer, 10 mM dNTP (dNTP mix), oligo dT primer, RNase inhibitor, and 25 mM MgCl_2_. Two microliters of each cDNA sample from the RT-PCR was amplified by PCR in 25 μL containing 10× buffer 2.5 μL, 25 mM MgCl_2_ 2.5 μL, and 10 pmol 0.75 μL primer. PCR was done using 10× Buffer for Taq polymerase (100 mM Tris-Cl pH 8.5, 400 mM KCl), 1 mM each dNTP, 50 mM MgCl_2_, upstream primer (5 μM), downstream primer (5 μM), and DNA template (less than 200 ng) SyBr Green). Primers were chemically synthesized using a DNA synthesizer (Bioneer, Daejeon, South Korea). Amplification conditions were denaturation at 94 °C for 3 min for the first cycle and for 30 s starting from the second cycle, annealing (68 °C for 30 s for β-actin, 69 °C for 30 s for inducible NO synthase [iNOS], and 68 °C for 30 s for cyclooxygenase-2 [COX-2] and interleukin [IL]-1β), and final extension at 72 °C for 7 min. The PCR products were resolved by electrophoresis on 1% or 2% agarose gel and stained with ethidium bromide. The primers used were based on GenBank data: β-actin (492 bp): 5′-TCC TTC GTT GCC GGT CCA CA-3′ (sense), 5′-GCT GTG TGT CAC AGA AGT CTC GAA CTC-3′ (anti-sense)/COX-2 (233 bp): 5′-TGT GAC TGT ACC CGG ACT GG-3′ (sense), 5′-TGC ACA TTG TAA GTA GGT GG-3′ (sense)/IL-1β (387 bp): 5′-TGC AGA GTT CCC CAA CTG GTA CAT C-3′ (sense), 5′-GTG CTG CCT AAT GTC CCC TTG AAT C-3′ (anti-sense).

### 3.9. Animal

Four-week-old male X-rats were individually housed in cages with alternating 12-h periods of light and dark, with free access drinking water and standard laboratory food for 2 weeks.

### 3.10. Induction of Urolithiasis

Experimental induction of urolithiasis was done using a modification of a previously published method by Itoh et al. Hyperoxaluria established using low or high concentrations of ethylene glycol (EG; Sigma-Aldrich) was used to induce urolithiasis. Thirty five 6-week-old male X-rats weighing 200–220 g were randomly divided into six groups containing six animals each: (1) acute control; (2) acute, 0.2% EG; (3) acute, 0.4% EG, (4) chronic control; (5) chronic, 0.2% EG; and (6) chronic, 0.4% EG. Sterilized water containing the particular concentration of EG was given orally every day (Table 6A). Rats were sacrificed by anesthesia using ether (Ducksan, Seoul, Korea) at 2 weeks and 4 weeks. 

### 3.11. Effect of QGB Extract on Urolithiasis

The QGB extract was used. Twenty four 6-week-old male X-rats (200–220 g) were randomly divided into four groups containing six animals each: (1) treatment with saline; (2) control (0.4% EG); (3) low-dose drug (50 mg/kg) + 0.4% EG; and (4) high-dose drug (100 mg/kg) + 0.4% EG. Saline, EG, and QGB extract treatment were administered by intraperitoneal (IP) injection (Table 6B). Rats were anesthesized using ether and sacrificed 2 weeks later by inhalation of CO_2_, and removed the kidneys and weighed the stones in kidneys. 

### 3.12. Statistical Analysis

All data are expressed as mean ± S.E.M. Values were analyzed by one-way analysis of variance (ANOVA) followed by Student-Newman-Keuls (S-N-K) test using the Statistical Package for the Social Sciences software package (SPSS Inc., Chicago, IL, USA). Significance was indicated by a *p*-value < 0.05 (in vitro) and 0.01 (in vivo). Values bearing different superscripts in the same column are significantly different. 

## 4. Conclusions

Activity-guided isolation of 80% acetone extract from the barks of QGB yielded several polyphenols. Those compounds showed potent activities on anti-oxidation and anti-inflammation in vitro. In addition, the QGB extract containing polyphenols showed strong anti-urolithiasis effect in vivo. In conclusion, catechins, condensed tannins and QGB extract showed potent anti-oxidative, anti-inflammatory, and anti-urolithiasis activities. These results show that QGB may be developed as a strong anti-inflammatory and anti-urolithiasis agents. 

## Figures and Tables

**Table 1:** IC_50_ values for DPPH radical scavenging activity from different parts of QG plants and from isolated compounds.

Extract	IC_50_ (μg/mL)	Compound	IC_50_ (μM)
Leave	19.15 ± 0.22	**1**	>100 ^h^
Branch	33.07 ± 0.34	**2**	>100 ^g^
Bark	10.58 ± 0.85	**3**	74.23 ± 0.39 ^f^
Wood	16.14 ± 0.80	**4**	43.37 ± 0.57 ^e^
l-ascorbic acid	6.04 ± 0.27	**5**	28.80 ± 1.02 ^d^
		**6**	15.10 ± 0.34 ^b^
		**7**	12.15 ± 0.18 ^a^
		l-ascorbic acid	27.41 ± 1.28 ^c^

Values are expressed as mean ± S.D. of three determinations. Values bearing different superscripts (a–g) in same columns are significantly different (*p* < 0.05).

**Table 2:** IC_50_ values for NBT/superoxide scavenging activity from different parts of QG plants and from isolated compounds.

Extract	IC_50_ (μg/mL)	Compound	IC_50_ (μM)
Leave	7.50 ± 0.47	**1**	>100 ^d^
Branch	20.07 ± 2.24	**2**	>100 ^c^
Bark	4.43 ± 0.16	**3**	>100 ^b^
Wood	7.07 ± 0.98	**4**	15.44 ± 1.44 ^a^
Allopurinol	1.08 ± 0.22	**5**	11.01 ± 0.16 ^a^
		**6**	7.21 ± 0.38 ^a^
		**7**	8.67 ± 0.22 ^a^
		Allopurinol	5.43 ± 0.40 ^a^

Values are expressed as mean ± S.D. of three determinations. Values bearing different superscripts (a–d) in same columns are significantly different (*p* < 0.05 NBT/superoxide scavenging activity).

**Table 3:** IC_50_ values of QGB compounds **1**–**7** on inhibition of NO production in RAW 264.7 macrophages.

Sample	NO Production Inhibitory Activity (μM)
**1**	>100 ^f^
**2**	63.99 ± 12.53 ^d^
**3**	9.14 ± 0.45 ^b^
**4**	>100 ^e^
**5**	14.47 ± 5.29 ^c^
**6**	7.07 ± 0.40 ^b^
**7**	1.44 ± 1.03 ^a^
l-NMMA	2.72± 0.80 ^a^

Values are expressed as mean ± S.D. of three determinations. Values bearing different superscripts (a–f) in same columns are significantly different (*p* < 0.05).

**Table 4:** Stone values of X-rats kidney tissue with ethylene glycol (EG) concentration.

Group No.	Treatment	Upper Pole	Mid Pole	Lower Pole	Average
Group 2	0.2% EG (acute)	35	57	38	44.33 ^a^
Group 3	0.4% EG (acute)	80	112.17	90	90.05 ^b^
Group 5	0.2% EG (chronic)	43.5	46.5	38	42.67 ^a^
Group 6	0.4% EG (chronic)	70.92	74.67	70.92	72.16 ^a,b^

Group 2: No. 7–11 (0.2% EG for 2 weeks) ^a^; Group 3: No. 12–18 (0.4% EG for 2 weeks) ^b^; Group 5: No. 25–30 (0.2% EG for 4 weeks) ^a^; Group 6: No. 31–35 (0.4% EG for 4 weeks) ^a,b^; Values bearing different superscripts (a, b) in same columns are significantly different (*p* < 0.01).

**Table 5:** Stone values of X-rats kidney section treated ethylene glycol (EG) and drug treatment.

Group No.	Treatment	Upper Pole	Mid Pole	Lower Pole	Average
B	EG	111.83	92.67	105.33	119.33
C	EG + low drug	5	4.67	2	2.33 *
D	EG + high drug	14	7.5	15	12.17 *

Group B: No. 7–12 (control: 0.4% of EG); Group C: No. 13–18 (EG + low dose drug for 2 weeks) *; Group D: No. 19–24 (EG + high dose drug for 2 weeks) *; The *p* values of mark [*] was less than 0.001.

**Table 6:** Experimental design of urolithiasis induction and anti-urolithiasis activity in the in vivo.

(A)		
Group No.	Day	Treatment
1	2 weeks	Normal Control (sterilized water)
2		0.2% EG, as acute model
3		0.4% EG, as acute model
4	4 weeks	Normal Control (sterilized water)
5		0.2% EG, as chronic model
6		0.4% EG, as chronic model
(**B**)		
**Group No.**	**Day**	**Treatment (IP)**
A	2 weeks	Normal Control (saline)
B		Control (0.4% EG)
C		0.4% EG + low drug
D		0.4% EG + high drug

## References

[1] Mandana Rodriguez A., Gausa Rull P. (1980). Therapeutic effects of Q*uercus* extract in urolithiasis. Arch. Esp. Urol..

[2] Sairam K., Scoffone C.M., Alken P., Turna B., Sodha H.S., Rioja J., Wolf J.S., de la Rosette J.J. (2012). CROES PCNL Study Group. Percutaneous nephrolithotomy and chronic kidney disease: Results from the CROES PCNL Global Study. J. Urol..

[3] Moriyama M.T., Miyazawa K., Noda K., Oka M., Tanaka M., Suzuki K. (2007). Reduction in oxalate-induced renal tubular epithelial cell injury by an extract from *Quercus* salicina Blume/*Quercus* stenophylla Makino. Urol. Res..

[4] Butterweck V., Khan S.R. (2009). Herbal medicines in the management of urolithiasis: Alternative or complementary?. Planta Med..

[5] Clark D.L., Connors B.A., Evan A.P., Handa R.K., Gao S. (2011). Effect of shock wave number on renal oxidative stress and inflammation. BJU Int..

[6] Nagata M., Takayama T., Mugiya S., Ohzono S. (2011). Pharmacotherapy for preventing calcium containing stone formation. Clin. Calcium.

[7] Sekkoum K., Cheriti A., Taleb S., Bourmita Y., Belboukhari N. (2011). Traditional phytotherapy for urinary diseases in Bechar district (south west of Algeria). Electron. J. Environ., Agric. Food Chem..

[8] Itoh Y., Yasui T., Okada A., Tozawa K., Hayashi Y., Kohri K. (2005). Preventive effects of green tea on renal stone formation and the role of oxidative stress in nephrolithiasis. J. Urol..

[9] Box E.O., Fujiwara K., Box E.O., Fujiwara K. (2015). Warm-Temperate Deciduous Forests: Concept and Global Overview. Warm-Temperate Deciduous Forests around the Northern Hemisphere.

[10] Tanouchi H., Sato T., Takeshita K. (1994). Comparative studies on acorn and seedling dynamics of four *Quercus* species in an evergreen broad-leaved forest. J. Plant Res..

[11] Young H.Y., Hae R.K. (2012). Ecophysiological responses of *Quercus gilva*, endangered species and Q. glauca to long-term exposure to elevated CO_2_ concentration and temperature. J. Ecol. Environ..

[12] Noshiro S., Sasaki Y. (2011). Identification of Japanese species of evergreen and *Quercus* and *Lithocarpus* (Fagaceae). IAWA J..

[13] Hamid H., Kaur G., Abdullah S.T., Ali M., Athar M., Alam M.S. (2005). Two new compounds from the galls of *Quercus* infectoria. with nitric oxide and superoxide inhibiting ability. Pharm. Biol..

[14] Indrianingsih A.W., Tachibana S., Dewi R.T., Itoh K. (2015). Antioxidant and α-glucosidase inhibitor activities of natural compounds isolated from *Quercus gilva* Blume leaves. Asian Pac. J. Trop. Biomed..

[15] Moon M., Baik J., Kim S., Jang W., Kim M., Lee N. (2009). Identification of antioxidative constituents from the branches of *Quercus gilva* Blume. J. Soc. Cosmet. Sci. Korea.

[16] Yamamoto M., Akita T., Koyama Y., Sueyoshi E., Matsunami K., Otsuka H., Shinzato T., Takashima A., Aramoto M., Takeda Y. (2008). Euodionosides A–G: Megastigmane glucosides from leaves of Euodia meliaefolia. Phytochemistry.

[17] Tachi Y., Kamano Y., Sawada J., Tanaka I., Itokawa H. (1976). Studies on the constituents of *Quercus* spp. VII. Triterpenes of *Quercus gilva* Blume (author’s transl). Yakugaku Zasshi.

[18] Itokawa H., Tachi Y., Kamano Y., Iitaka Y. (1978). Structure of gilvanol, a new triterpene isolated from *Quercus gilva* Blume. Chem. Pharm. Bull..

[19] Zhong X.N., Ide T., Otsuka H., Hirata E., Takeda Y. (1998). (+)-Isolarisiresinol 3a-*O*-sulphate from leaves of Myrsine seguinii. Phytochemistry.

[20] Jiang Z.H., Tanaka T., Sakamoto M., Jiang T., Kouno I. (2001). Studies on a medicinal parasitic plant: Lignans from the stems of Cynomorium songaricum. Chem. Pharm. Bull..

[21] Ruberto G., Renda A., Daquino C., Amico V., Spatafora C., Tringali C., Tommasi N.D. (2007). Polyphenol constituents and antioxidant activity of grape pomace extracts from five sicilian red grape cultivars. Food Chem..

[22] Nonaka G., Nishioka I. (1982). Tannins and related compounds. VII. Phenylpropanoid-substituted epicatechins, cinchonains from Cinchona succirubra. (1). Chem. Pharm. Bull..

[23] Fan J., Ding X., Gu W. (2007). Radical-scavenging proanthocyanidins from sea buckthorn seed. Food Chem..

[24] Cai Y., Evans F.J., Roberts M.F., Phillipson J.D., Zenk M.H., Gleba Y.Y. (1991). Polyphenolic compounds from Croton lechleri. Phytochemistry.

[25] Kehrer J.P. (1993). Free radicals as mediators of tissue injury and disease. Crit. Rev. Toxicol..

[26] Moskovitz J., Yim M.B., Chock P.B. (2002). Free radicals and disease. Arch. Biochem. Biophys..

[27] Lin M.Y., Yen C.L. (1999). Antioxidative Ability of Lactic Acid Bacteria. J. Agric. Food Chem..

[28] Hatano T., Yasuhara T., Yoshihara R., Agata I., Noro T., Okuda T. (1990). Effects of interaction of tannins with co-existing substances. VII: Inhibitory effects of Tannins and related polyphenols on xanthine oxidase. Chem. Pharm. Bull..

[29] Parejo I., Viladomat F., Bastida J., Rosas-Romero A., Flerlage N., Burillo J., Codina C. (2002). Comparison between the radical scavenging activity and antioxidant activity of six distilled and nondistilled mediterranean herbs and aromatic plants. J. Agric. Food Chem..

[30] Kharitonov S.A., Yates D., Robbins R.A., Logan-Sinclair R., Shinebourne E.A., Barnes P.J. (1994). Increased nitric oxide in exhaled air of Asthmatic Patients. Lancet.

[31] Cals-Grierson M.M., Ormerod A.D. (2004). Nitric Oxide Function in the Skin. Nitric Oxide.

[32] Akdeniz N., Aktaş A., Erdem T., Akyüz M., Özdemir Ş. (2004). Nitric oxide levels in atopic dermatitis. Pain Clin..

[33] Epe B., Ballmaier D., Roussyn I., Briviba K., Sies H. (1996). DNA damage by peroxynitrite characterized with DNA repair enzymes. Nucleic Acids Res..

[34] Hobbs A.J., Higgs A., Moncada S. (1999). Inhibition of nitric oxide synthase as a potential therapeutic target. Annu. Rev. Pharmacol. Toxicol..

[35] Sautebin L. (2000). Prostaglandins and nitric oxide as molecular targets for anti-inflammatory therapy. Fitoterapia.

[36] Kim J.Y., Kim Y.S., Chang I.H., Kim T.H., Kim H.R. (2011). Interleukin-1β, calcium-sensing receptor, and urokinase gene polymorphisms in Korean Patients with urolithiasis. Korean J. Urol..

[37] Wang S., Wang X., Wu J., Lin Y., Chen H., Zheng X., Zhou C., Xie L. (2012). Association of vitamin D receptor gene polymorphism and calcium urolithiasis in the Chinese Han population. Urol. Res..

[38] Chou Y.H., Woon P.Y., Chen W.C., Hsu Y.W., Chang J.M., Hwang D.Y., Chiu Y.C., Kuo H.C., Chang W.P., Hou M.F (2011). A genetic polymorphism (rs17251221) in the calcium-sensing receptor gene (CASR) is associated with stone multiplicity in calcium nephrolithiasis. PLoS ONE.

[39] Canaff L., Hendy G.N. (2005). Calcium-sensing receptor gene transcription is up-regulated by the proinflammatory cytokine, interleukin-1β role of the NF-κB pathway and κB elements. J. Biol. Chem..

[40] Kandhare A.D., Patil M.V., Bodhankar S.L. (2015). l-Arginine attenuates the ethylene glycol induced urolithiasis in ininephrectomized hypertensive rats: Role of KIM-1, NGAL, and NOs. Ren. Fail..

[41] Mittal R.D., Bid H.K., Manchanda P.K., Kapoor R. (2007). Association of interleukin-1β gene and receptor antagonist polymorphisms with calcium oxalate urolithiasis. J. Endourol..

[42] Nakada S.Y., Jerde T.J., Jacobson L.M., Saban R., Bjorling D.E., Hullett D.A. (2002). Cyclooxygenase-2 expression is up-regulated in obstructed human ureter. J. Urol..

[43] Grases F., Prieto R.M., Gomila I., Sanchis P., Costa-Bauzá A. (2008). Phytotherapy and renal stones: The role of antioxidants. A pilot study in wistar rats. Urol. Res..

[44] Grases F., Prieto R.M., Fernandez-Cabot R.A., Costa-Bauza A., Tur F., Torres J.J. (2015). Effects of polyphenols from grape seeds on renal lithiasis. Oxid. Med. Cell. Longev..

[45] Feelisch M., Stamler J. (1996). Methods in Nitric Oxide Research.

